# Exposure to social bots amplifies perceptual biases and regulation propensity

**DOI:** 10.1038/s41598-023-46630-x

**Published:** 2023-11-24

**Authors:** Harry Yaojun Yan, Kai-Cheng Yang, James Shanahan, Filippo Menczer

**Affiliations:** 1 The Media School, Indiana University, Bloomington, IN 47405 USA; 2 Luddy School of Informatics, Computing, and Engineering, Indiana University, Bloomington, IN 47408 USA; 3 Observatory on Social Media, Indiana University, Bloomington, IN 47408 USA

**Keywords:** Human behaviour, Information technology

## Abstract

Automated accounts on social media that impersonate real users, often called “social bots,” have received a great deal of attention from academia and the public. Here we present experiments designed to investigate public perceptions and policy preferences about social bots, in particular how they are affected by exposure to bots. We find that before exposure, participants have some biases: they tend to overestimate the prevalence of bots and see others as more vulnerable to bot influence than themselves. These biases are amplified after bot exposure. Furthermore, exposure tends to impair judgment of bot-recognition self-efficacy and increase propensity toward stricter bot-regulation policies among participants. Decreased self-efficacy and increased perceptions of bot influence on others are significantly associated with these policy preference changes. We discuss the relationship between perceptions about social bots and growing dissatisfaction with the polluted social media environment.

## Introduction

Social bots are social media accounts that are controlled at least in part by software on social media. They can be purchased at low cost^1^ and operated with various degrees of automation. By posting content and interacting with people, some bots can emulate and deceive real social media users, posing a threat to our social and political life^2^. Social bots have been used to disseminate fake news^3^ and inflammatory information^4,5^, exploit the private information of users^6^, sway public attention on controversial topics^7–9^, and create false public support for political and commercial gain^1,10,11^, especially during major political events such as elections^12–14^. While the severity of the threats posed by bots is still debated^15^, research has demonstrated critical consequences of bot exposure^16,17^.

Public awareness of social bots has been on the rise and a majority of Americans believe that bots have negative effects on the public^18,19^. Yet, public perceptions of bots are under-studied^20^. A consensus on the definition of social bots is lacking; people appear to use the term to refer to a variety of entities, from fake profiles and spammers to fully-automated accounts^21^. This ambiguity provides social media users with a scapegoat for their unpleasant online experiences^22^. For example, one may reject accounts with opposing political views by labeling them as bots^17,23^. Such a confirmation bias is only one of many perceptual biases on which users rely when making judgments about online interactions^24^. These biases have a strong evolutionary foundation^25^ but make us vulnerable to manipulation^26^.

Here we report on two experiments designed to investigate perceptual biases regarding social bots and how they are exacerbated by exposure to bots. In the experiments, participants are instructed to distinguish bot-like social media profiles from authentic users; no feedback is provided. Participants answer questions regarding their perceptions about bots before and after the bot exposure. While these two experiments conceptually replicate each other, they differ slightly in their design. In *Experiment I*, participants were shown profiles containing a mix of non-political and political profiles. Half of the participants were assigned to a condition where the ambiguity between humans and bots was low, while the other half were placed in a condition with high ambiguity. In *Experiment II*, all participants viewed the same set of political profiles, and the overall level of ambiguity between humans and bots was comparable to the high-ambiguity condition in Experiment I. Further methodological details can be found in the “Materials and Methods” section.

Consequently, combining the results of these two experiments allowed for the creation of three analytical conditions: (1) profiles with low human-bot ambiguity and mixed content, (2) profiles with high human-bot ambiguity and mixed content, and (3) profiles with high human-bot ambiguity and political content. By comparing the results from these three conditions, we can also shed light on the impact of varying levels of human-bot ambiguity and explore any differential effects resulting from the presence of political bots as opposed to non-political bots.

We focus on the effects of bot exposure on three perceptions by social media users: estimated prevalence of bots, perceived influence of bots on themselves and others, and assessment of one’s own ability to recognize bots. Theories of cognitive biases predict that these perceptions are intertwined and often inaccurate^25,27–30^. More importantly, manipulations of these perceptions drive behavioral changes such as the adoption of different policies^31,32^. Therefore, we also survey preferences for more or less strict countermeasures. To examine the effects of bot exposure, we compare the same set of measures before and immediately after exposure. We find that perceptual biases regarding bots exist and can be amplified by simply raising awareness of the potential threat they pose.

Quantifying perceptual biases about social bots may also help improve the design of machine learning algorithms to detect them^33–35^. These algorithms depend on labeled examples of bot accounts, which are often identified by human annotators^36^. The biases of annotators can therefore propagate through the pipeline and affect downstream tasks. As a few studies have already revealed perceptual biases in human-bot interactions^17,23^, more research is needed.

While policy efforts about social bots are still in the nascent stage^37^, our findings about bot regulation preferences suggest that policymakers should be cautious when interpreting public opinion data. As demonstrated in this study, public sentiment toward the perceived threats posed by bots can exhibit reactionary and irrational patterns. Recognizing the dynamic and evolving nature of public sentiment can assist policymakers in crafting more effective regulations and strategies concerning social bots.

## Results

### Overestimation of bot prevalence

The prevalence of bots has been at the center of public discussion since it is closely related to user experience and the financial value of social media platforms. Consensus on a number is unlikely because scholars and researchers cannot agree on the definition of bots^37^ and detection is technically challenging^21^. Different stakeholders have provided rather disparate estimates: Twitter’s Annual Report in 2021 recognized that 5% of accounts might be spam or false^38^; Elon Musk claimed the number to be as high as 20% (youtube.com/watch?v=CnxzrX9tNoc, 3′ 08″–3′ 30″); these estimates are tainted by potential conflicts of interest^39^. A scholarly estimate of automated accounts on Twitter ranged between 9% and 15% in 2017^40^.

Rather than focusing on the *actual* number of bots, we are interested in investigating prevalence as *perceived* by the general public. People commonly have perceptual biases about the prevalence of social phenomena. For example, they tend to believe that a small sample is representative of a larger population^27^. Such a perceptual bias becomes more prominent and is easily manipulated by media messages when the judged matter is deemed undesirable^41–43^. Therefore, we expect participants to overestimate the prevalence of bots and that such bias would be further magnified after experimental exposure to bots (see “Materials and Methods” Section).

**Figure 1:**
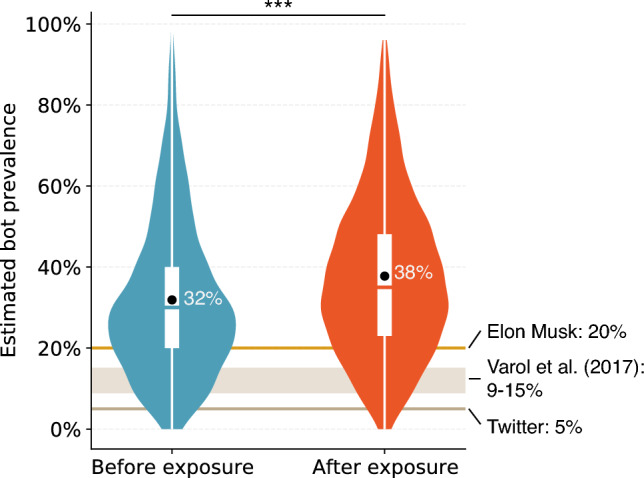
Overestimation of bot prevalence. These violin plots show the distributions of bot prevalence estimates by participants before and after exposure to bots impersonating humans; the difference is significant ($p<0.001$). The black dots indicate the mean values; the box plots show the 25th, 50th, and 75th percentiles. We also show the estimates of inauthentic and spam accounts by Twitter and Elon Musk together with the estimated prevalence of automated accounts by Varol et al.^40^. See the main text for details.

Before exposure to bots in the recognition task, participants report that on average 31.9% (SD 18.9%, Median 30.0%) of social media accounts are bots, which is substantially larger than the estimates provided by Twitter, Varol et al.^40^, and even Musk. This result suggests that participants tend to overestimate the prevalence of bot accounts. After exposure to bots, the average estimated prevalence goes up to 37.8% (SD 19.2%, Median 35.0%). Figure 1 shows the distributions of the estimates before and after the recognition tasks and the significant increase after exposure (Wilcoxon signed rank test *V* = 99,448, $p< 0.001$).

### Misjudged self-efficacy in bot recognition

While people tend to overestimate the prevalence of threats, they may or may not believe they are capable of mitigating such threats. On the one hand, it is beneficial for people to believe in their ability, since such self-efficacy can promote task performance^44–47^. On the other hand, self-assessments of expertise, such as information literacy^48^, can be inaccurate and inflated^28,49–51^. To investigate whether self-efficacy in the bot-recognition task is reliable, we asked participants to assess their ability to identify bots before and after exposure and tested the association between their self-assessments and their actual accuracy (see “Materials and Methods” Section).

**Figure 2:**
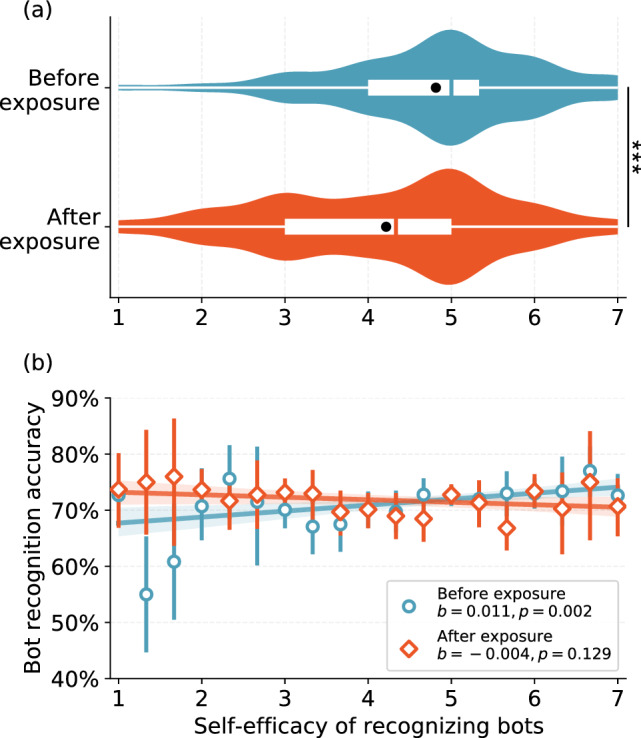
Self-efficacy and bot recognition accuracy. (**a**) The violin plots show the distributions of self-reported bot-recognition efficacy by participants before and after exposure to bots impersonating humans; the difference is significant ($p<0.001$). Self-efficacy is in the range 1–7 where a larger value indicates higher confidence. The black dots indicate the mean values; the box plots show the 25th, 50th, and 75th percentiles. (**b**) Relation between participant self-efficacy and actual bot-recognition accuracy before and after exposure. The coefficients estimated from the linear regressions and the *p*-values are annotated.

We find that the majority of participants possess a somewhat high bot-recognition self-efficacy before exposure to bots (Mean 4.8, SD 1.2, Median 5.0 on a 7-point Likert scale). After exposure to bots, participants become less confident (Mean 4.2, SD 1.2, Median 4.3). Bot exposure causes on average a significant decrease in self-efficacy among participants (paired *t*-test $t = -10.3, p < 0.001$), as shown in Fig. 2a.

We used individual-level regression analysis to assess the relationship between the self-efficacy of participants and their actual accuracy in recognizing bots. The results are reported in Fig. 2b and Table 1. We find that pre-exposure self-efficacy is positively associated with performance ($b=0.011, S.E.=0.003, p=0.002$), although the effect size is rather minimal (adjusted $R^2 = 0.009$). The post-exposure self-efficacy, on the other hand, is not significantly correlated with performance ($b=-0.004, S.E.=0.003, p=0.129$), showing that exposure to bots further distorts the self-assessment of the participants. An additional regression shows that participants who report a larger *improvement* in their self-efficacy perform significantly worse in the bot recognition task ($b =-0.009, S.E.=0.002, p<0.001, R^2=0.011$; see full model results with control variables in Table 1). Overall, these results suggest that self-efficacy is not a reliable predictor for the actual ability to recognize bots, and exposure to bots further exacerbates this unreliability.

**Table 1:** Generalized linear models of self-efficacy predicting performance in the bot-recognition task.

	Before exposure	After exposure	Changes
(Intercept)	1.29***	1.28***	1.26***
High-ambiguity mixed bots^a, c^	− 0.47***	− 0.47***	− 0.47***
High-ambiguity political bots^b, c^	− 0.36***	− 0.35***	− 0.32***
Age	− 0.08***	− 0.09***	− 0.09***
Education	− 0.01	− 0.01	− 0.01
Independent^d^	− 0.02	− 0.02	− 0.02
Republican^d^	− 0.12*	− 0.12*	− 0.12*
Self-efficacy			
Before exposure	0.05*		
After exposure		− 0.01	
Change scores			− 0.04*
$R^2$	0.094	0.089	0.093

The dependent variable is the proportion of accurate answers out of twenty trials. We also carried out the regression at the trial level, and the results are consistent except for the self-efficacy changes (the last column), where the association with the actual accuracy yields $$p=.051. $$

$${^{*} } p<.05; {^{***} }p<.001$$.

We use beta distribution as the link function

^a^The second condition of *Experiment I*

^b^*Experiment II*

^c^Low-ambiguity mixed bots (i.e., the first condition of *Experiment I*) as the reference group

^d^Democrat as the reference group.

### Gap in perceived bot influence on others vs. self

**Figure 3:**
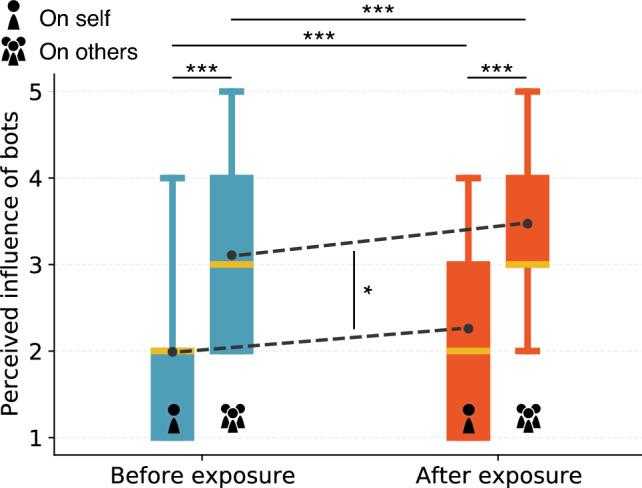
Stronger perceived bot influence on others than on selves. The box plots show perceived influence of bots on others versus on participants themselves before and after exposure to bots impersonating humans. The magnitude of the perceived influence is in the 1–5 range. The perceived influence of bots on others is significantly stronger than on participants themselves, indicating third-person perceptions, both before and after exposure ($p<0.001$). The perceived influence of bots on self and others both significantly increase after exposure ($p<0.001$). The gap between perceived influence on others and on participants themselves is slightly but significantly larger after exposure ($p=0.021$).

People commonly assume stronger media effects on others than on themselves when facing adversarial media messages. This so-called “third-person perception” (TPP)^29^ is moderate but robust in the context of mass media messaging^52–54^. The perceptual gap associated with TPP can be further magnified when the judged matter is threatening to existing social norms^55^. Surveys have also reported on TPP in new media environments, such as internet pornography^56^, Facebook trolls^57^, and online fake news^58^. However, the change in an individual user’s TPP caused by direct interactions with media content is less studied^59^. Following these previous studies, we hypothesize that TPP could also be observed in the context of interactions with social bots and amplified after exposure.

We measure perceived influence on a five-point scale, where a larger value indicates a stronger effect (see “Materials and Methods” Section). As shown in Fig. 3, participants perceive stronger bot influence on others (Mean 3.1, SD 0.9) than on themselves (Mean 2.0, SD 0.9) before exposure to bots. The difference is significant (paired *t*-test $t = 31.6, p < 0.001$). After exposure, we observe significant increases in the perceived influence on selves ($t = 9.4, p < 0.001$) as well as on others ($t = 11.4, p < 0.001$). However, TPP persists (influence on others: Mean 3.5, SD 1.0; on self: Mean 2.3, SD 1.0; gap: $t = 31.7, p < 0.001$). In fact, the gap between the perceived influence of bots on others versus on selves widens slightly after exposure: $t = 2.3, p = 0.021$. These results suggest that exposure to bots amplifies not only the perceived bot influence but also the TPP bias of the participants.

### Propensity for more stringent bot regulation

**Figure 4:**
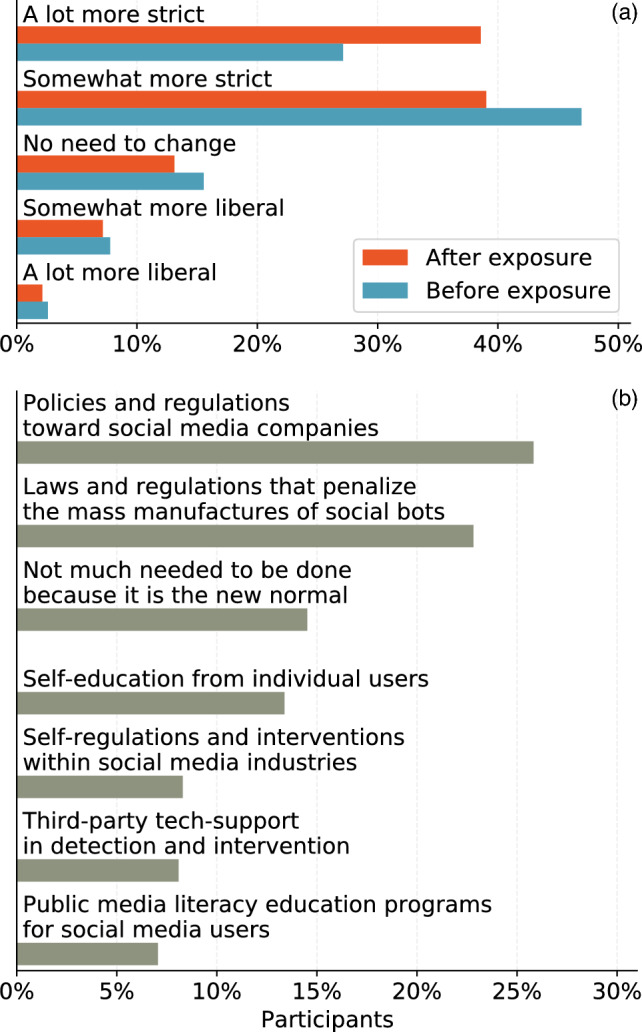
Preferences for social bot countermeasures. (**a**) Percentages of participants preferring more strict or more liberal restrictions before and after exposure to bots. (**b**) Percentages of participants who rank different countermeasures as their top choices.

We asked the participants about their views regarding regulations and other countermeasures toward social bots (see “Materials and Methods” Section). The results are presented in Fig. 4a. We find that the preference for stricter regulations among participants is significantly amplified by exposure to bots ($t=5.1, p<0.001$). The group favoring the strictest measures grows from 27.1% to 38.6%.

**Table 2:** Ordinary least-square models predicting changes of policy restrictions.

Predictors	Model I^1^		Model II^2^	
	*B*	*p*	*B*	*p*
(Intercept)	0.05	0.443	0.04	0.478
Age	− 0.04	0.33	− 0.04	0.338
Education	− 0.06	0.112	− 0.06	0.108
Independent^a^	− 0.06	0.535	− 0.03	0.56
Republican^a^	− 0.12	0.259	− 0.11	0.302
Change scores^b^				
Perceived prevalence	0.06	0.153	0.04	0.308
Self-efficacy	**− 0.11**	**0.005**	**− 0.1**	**0.008**
Third-person perceptions	0.07	0.073		
Perceived influence on self			− 0.00	0.988
Perceived influence on others			**0.10**	**0.015**
$R^2$/ $R^2$ adjusted	0.03/0.02		0.034/0.022	

Significant values with *p* below 0.05 are in [bold].

^1^Model I includes the discrepancy between perceived influences on others and on selves as a single independent variable to measure the effect of third-person perceptions.

^2^Model II includes the perceived influence of bots on others and on selves as separate independent variables.

^a^Democrat is the reference group.

^b^Change scores are the differences between pre- and post-exposure measures.

We also investigate whether changes in regulation preference are predicted by the changes in the three measures explored in previous sections while controlling for demographic factors. The overall model contributions to the change in preference are small ($R^2<0.05$, see Table 2). However, we find a significant association between the decrease in bot-recognition self-efficacy and the dependent variable ($b =-0.11, p=0.005$), whereas the change in perceived prevalence is not a significant predictor. While TPP (the perceptual gap between bot influence on others and on self) is not a significant predictor, the increase in perceived influence on others caused by exposure is significantly associated with the preference toward more stringent regulation ($b=0.10, p<0.015$).

We also asked participants to rank their preferences among countermeasures targeting different stakeholders after exposure to bots (see “Materials and Methods”). The results are shown in Fig. 4b. Overall, a majority of participants rank top-down options, such as legislative regulations targeting social media platforms (25.8%) and penalizing bot operators (22.8%), as their preferred policies. The countermeasures that have received the most attention from platforms and researchers, including company self-regulation (8.3%), third-party support (8.1%), and media literacy campaigns (7.1%), have less support. A substantial portion of the participants (14.5%) are in favor of accepting the existence of bot manipulation as the “new normal”.

## Discussion

Our experimental design has some limitations. First, the participants were asked the same questions twice. Since we did not have a separate control group, we cannot exclude the potential confounding effect of sequential testing. Second, the profiles selected in the experiments may not be representative of real-life bot exposure. Third, two of our findings regarding third-person perception changes and regulation preferences are statistically significant but have small effect sizes.

With these caveats, this study demonstrates how exposure to social bots significantly distorts perceptions of bot prevalence and influence. First, we find that potentially inflated estimates about bot prevalence on social media are further amplified after bot exposure. According to the “law of small numbers” bias^27^, this overestimation can be attributed in part to participants extrapolating from the few examples in the experiment to the entire social media environment. The susceptibility of prevalence estimates to experimental manipulations also underscores the effects of media interactions on perceptions of social reality. For example, heavy consumers of TV entertainment, which frequently features violent stories and scenes, tend to exaggerate the prevalence of violence in the real world^43,60^. Just as people who perceive the world as more dangerous because of TV viewing develop a strong “mean-world” sentiment^61^, the overestimation of social bots may exemplify dissatisfaction with a polluted social media environment^22^.

Second, our results suggest that the participants generally have an optimistic but unreliable assessment of their own ability to recognize social bots. Prior to bot exposure, participants tended to express confidence in their bot-recognition skills. However, exposure to bots with no feedback created self-doubt. On one hand, participants may have encountered greater difficulty than anticipated in the bot recognition tasks, since our stimuli included some highly ambiguous accounts (see “Materials and Methods” Section: Profile Selection). On the other hand, this result underscores the malleability of one’s assessments regarding their own ability to detect bots and the potential recency bias of such judgments. While self-efficacy is positively correlated with actual bot recognition performance before bot exposure^44^, the effect size is small, and the correlation is weaker after exposure. Furthermore, those who report larger improvements after the bot recognition task tend to perform worse—exposure actually raises the susceptibility to bot deception among over-confident users. These findings are consistent with the Dunning-Kruger effect^28,51^ about the inability to objectively assess one’s own expertise.

Third, participants believe that other social media users are more vulnerable to bot influence than themselves, consistent with the third-person perceptions observed in other contexts involving negative media messages^52^. Such a self-other perceptual gap can in part be explained by the typical egotistic bias^62,63^. Bot exposure widens the perceptual gap by weakening one’s notion of self-immunity and to a greater extent by elevating the perceived vulnerability of others.

Finally, priming social media users to the threats posed by bots could unintentionally exacerbate a culture of distrust. After bot exposure, the majority of participants express preferences for regulations that target bot operators and social media companies over other options. This is consistent with a growing demand for governmental oversight of social bots^37^ and may reflect an increasing public distrust towards social media companies^64^. Our analysis suggests that the public support for more top-down policies may be due to uncertainty about one’s vulnerability to bot manipulation and fear of bot influence on others. The support for more stringent policy is susceptible to experimental manipulation and can be seen to stem from common cognitive biases, indicating that such policy preferences are not entirely rational. Regulating bots also raises First Amendment issues in the U.S.^65^. We believe that regulations may play a positive role in countering social media manipulation, but only in combination with other interventions^66^, such as information literacy and continued development of bot detection systems.

While our current study has provided insights into the immediate effects of exposure to social bots, it is possible that these effects could be reactive and temporary. However, it is essential to recognize that social bots have long been and will continue to be an integral component of social media platforms. As the long-term effects of social media gradually unfold^67,68^, there is a growing interest in understanding the enduring impact of bots. To address this important aspect, we are actively considering the development of multi-wave experiments and public opinion tracking polls. These future studies will allow us to explore how the evolving landscape of social bots, possibly powered by state-of-the-art artificial intelligence technologies^69^, affects user behavior, policy preferences, and other relevant outcomes over time.

## Materials and methods

*Bot recognition task* We conducted two experiments with the same pre-test questionnaire, followed by slightly different bot recognition tasks and post-test questionnaires. After finishing the pre-test questionnaires, participants were instructed to view 20 Twitter user profiles, half of which were bot-like and the other half were real users (the selection process is explained below). Participants were directed to the actual profiles through links to twitter.com. After viewing each profile, participants were asked to label it as human or bot.

*Profile selection* Following Yan et al.^17^, we relied on Botometer scores^36^ and expert coding for profile selection. Botometer is a widely used machine-learning tool for bot detection (botometer.org). It generates a score for each profile ranging from 0 to 5, with higher scores indicating more likely automated accounts. Scores close to 2.5 suggest high ambiguity. We started with an initial profile pool that consisted of randomly sampled 28,558 followers of U.S. congresspeople from both Republican and Democratic parties. We then sampled a total of 1,561 profiles with low (below 0.1) or high (above 4.9) bot scores, and 785 ambiguous profiles (bot scores around 2.5). During the final selection, expert coding by two authors was used to label the political nature and bot-likeness of the profiles independently. The coders placed particular emphasis on profiles with high ambiguity, as they presented challenges for Botometer. The expert coders underwent training to evaluate multiple profile heuristics systematically. Specifically, they considered factors such as the consistency of screen names and handles, the authenticity of profile and background pictures, the profile descriptions, the numbers of followers and friends, total tweet counts, tweet frequency, the percentage of original tweets and retweets, as well as the diversity and content authenticity of tweets in the timelines. Notably, two expert coders yielded fully consistent results. The final profile pool consisted of a total of 40 profiles that included even portions of political and non-political profiles, bot-like and authentic users, and low-ambiguity and high-ambiguity profiles. In addition to bot scores and expert coding, the human/bot classification of the 40 profiles was additionally corroborated by a crowdsourcing strategy, which used the majority of answers from a partisanship-balanced subsample of participants. All of the accounts were still active immediately after the experiment.

*Experiment I* used a mixed between/within-subject design. Participants ($N = 308$) were assigned to either a low-ambiguity or a high-ambiguity condition. In the low-ambiguity condition, profiles were selected with bot scores below 0.1 or above 4.9. In this condition, the human/bot labels generated by the three approaches mentioned above were fully consistent. In the high-ambiguity condition, half of the selected profiles had ambiguous scores (close to 2.5); the profiles in this condition were labeled by expert coding and crowdsourcing. In both conditions, only half of the profiles exhibited clear political partisanship.

*Experiment II* adopted a pure within-subject design: all participants ($N = 656$) viewed the same 20 profiles, all of which exhibited clear partisanship (e.g., including identity markers such as “#Republican” or “#VoteBlue”), and half of which had ambiguous scores. This experiment included additional policy-related questions in the post-test questionnaire. Analyses of perceptions about bot prevalence, self-efficacy, and bot influence are based on merged data from the two experiments, while the policy analysis is based on Experiment II only.

*Sampling* This study enlisted participants from Amazon Mechanical Turk (MTurk). Previous research has indicated that samples recruited from MTurk can effectively capture participants with a wide range of backgrounds^70^. However, these samples tend to over-represent digitally active individuals and exhibit higher levels of digital literacy^71^. While this may constrain the generalizability of MTurk samples to other research domains, we consider active internet and social media users as the target population in the context of the current study.

*Prevalence of bots* We measured the perceived prevalence of bots before and after the bot recognition task with the same question: “According to your estimation, what percentage of accounts on social media do you think are social bots?” Participants answered the questions with a slider ranging from 0 to 100 percent.

*Self-efficacy* We measured the self-efficacy in recognizing bot profiles by asking participants to what extent they agree with the following three statements: (1) “I will recognize social bots if I encounter them”; (2) “I believe I can succeed at telling social bots apart from real users”; and (3) “When facing social bots that highly resemble regular users, I can still find clues to weed them out.” The answers included seven options, ranging from “Strongly disagree” to “Strongly agree.” The same questions were asked before and after the bot recognition task (Cronbach $\alpha = 0.88 \text { and } 0.93$, respectively).

*Third-person perception* We measured the TPP of bots by asking two questions about the extent to which participants thought that social bots might have influenced them and average social media users. Participants were given five-point options, ranging from “Not at all” to “A lot,” to answer each question. We analyzed the two answers and their discrepancy.

*Countermeasure preferences* We asked participants before and after the bot recognition task: “If we were to restrict the production and use of bots on social media, what kind of changes would you like to see?” with five-point options ranging from “A lot more liberal” to “A lot more strict.” We also asked participants to rank seven specific countermeasures from the most preferred to the least (see wordings in Fig. 4b).

*Control variables* Our analysis included controls for participants’ partisanship, age, and education level (see summary of demographic information in Table 3). We initially considered self-reported variables such as the frequency of Twitter usage ($M = 3.16, SD = 1.28$) and past encounters with social bots ($M = 3.04, SD = 1.04$) as potential control variables. Participants were asked two questions: “How often do you check Twitter?” and “How many times do you think you have encountered social bots on social media before?” Their responses were measured on a five-point scale ranging from “never” to “very frequently.” However, the preliminary analysis suggested that self-reported Twitter usage frequency ($r= -0.02, p =0.508$) and prior encounters with bots ($r= 0.02, p =0.479$) did not significantly predict performance in bot recognition tasks. Consequently, these variables were not included in the final analysis.

**Table 3:** Demographic information.

	Overall	Experiment I	Experiment II
	(N = 964)	(n = 308)	(n = 656)
Partisanship			
Democrat	41.26%	40.39%	41.67%
Republican	23.28%	23.78%	23.03%
Independents	35.44%	38.83%	35.26%
Age (Mean)	34.92	34.38	35.17
Age (SD)	11.45	11.06	11.62
Education			
< College	21.16%	35.71%	14.39%
College or equivalent	50.51%	49.03%	51.21%
> College	28.31%	15.25%	34.45%

*Experimental protocols* The research methods and materials were approved by the Institutional Review Board at Indiana University-Bloomington under Protocol #1811295947 prior to the experiments. Informed consent was obtained from all participants before they proceeded to participate in the experiments. All methods were performed in accordance with the relevant guidelines and regulations.

## Data Availability

The dataset used in the current study is available in the GitHub repository at https://github.com/osome-iu/bot_perception_bias.
